# Targeting the Wnt/β-Catenin Signaling Pathway in Liver Cancer Stem Cells and Hepatocellular Carcinoma Cell Lines with FH535

**DOI:** 10.1371/journal.pone.0099272

**Published:** 2014-06-18

**Authors:** Roberto Gedaly, Roberto Galuppo, Michael F. Daily, Malay Shah, Erin Maynard, Changguo Chen, Xiping Zhang, Karyn A. Esser, Donald A. Cohen, B. Mark Evers, Jieyun Jiang, Brett T. Spear

**Affiliations:** 1 Department of Surgery, University of Kentucky, Lexington, Kentucky, United States of America; 2 Department of Physiology, University of Kentucky, Lexington, Kentucky United States of America; 3 Department of Microbiology, Immunology & Molecular Genetics, University of Kentucky, Lexington, Kentucky, United States of America; 4 Markey Cancer Center, University of Kentucky, Lexington, Kentucky, United States of America; National University of Singapore, Singapore

## Abstract

Activation of the Wnt/β-catenin pathway has been observed in at least 1/3 of hepatocellular carcinomas (HCC), and a significant number of these have mutations in the β-catenin gene. Therefore, effective inhibition of this pathway could provide a novel method to treat HCC. The purposed of this study was to determine whether FH535, which was previously shown to block the β-catenin pathway, could inhibit β-catenin activation of target genes and inhibit proliferation of Liver Cancer Stem Cells (LCSC) and HCC cell lines. Using β-catenin responsive reporter genes, our data indicates that FH535 can inhibit target gene activation by endogenous and exogenously expressed β-catenin, including the constitutively active form of β-catenin that contains a Serine37Alanine mutation. Our data also indicate that proliferation of LCSC and HCC lines is inhibited by FH535 in a dose-dependent manner, and that this correlates with a decrease in the percentage of cells in S phase. Finally, we also show that expression of two well-characterized targets of β-catenin, Cyclin D1 and Survivin, is reduced by FH535. Taken together, this data indicates that FH535 has potential therapeutic value in treatment of liver cancer. Importantly, these results suggest that this therapy may be effective at several levels by targeting both HCC and LCSC.

## Introduction

Hepatocellular carcinoma (HCC), the most common liver cancer, is the fifth most common cancer and the third highest cause of cancer-related mortality worldwide [1]–[2]. The alarming rise in HCC incidence in Europe and North America in recent years is related mainly to hepatitis C virus infection, although other factors such as excessive alcohol consumption and obesity also contribute to this increase [3]. The etiology of HCC is complex and involves numerous genetic and epigenetic alterations and the disruption of various signaling pathways including the Wnt/β-catenin, Ras/Raf/MAPK, PI3K/AKT/mTOR, HGF/c-MET, IGF, VEGF and PDGF pathways. Among these, the Wnt/β-catenin pathway is considered among the most difficult to inhibit [4]. Currently, few chemical agents targeting the Wnt/β-catenin pathway are available or under investigation [5].

Activation of the canonical Wnt/β-catenin pathway involves the binding of Wnt proteins to cell surface Frizzled receptors and LRP5/6 co-receptors. In the absence of Wnt proteins, much of the cellular β-catenin is bound to E-cadherin on the cell membrane. Cytosolic β-catenin is constitutively phosphorylated at specific serine residues by an enzymatic complex that includes adenomatous polyposis coli (APC), Axin, and the kinases glycogen synthase kinase-3β (GSK-3β) and casein kinase I, marking it for ubiquitin-mediated proteolysis. Under these conditions, the TCF/LEF transcription factors are bound to their cognate DNA recognition elements along with members of the Groucho family of co-repressors, insuring the transcriptional silencing of β-catenin target genes. Engagement of Wnt proteins with the Frizzled receptor activates the Dishevelled protein, resulting in the dissociation of the cytosolic destructive complex and inhibition of GSK-3β. This leads to the stabilization and accumulation of cytoplasmic β-catenin, which then enters the nucleus, binds TCF/LEF proteins and leads to the subsequent dissociation of groucho co-repressors, recruitment of the coactivator p300 and activation of β-catenin target genes [6]–[9]. Many of the β-catenin targets, including Cyclin D1, c-myc and Survivin, promote cell cycle progression and inhibit apoptosis [10]–[12]. Consistent with this data, activation of the Wnt/β-catenin pathway is seen in a variety of cancers, including HCC. Aberrant activation of the Wnt/β-catenin pathway has been observed in at least 1/3 of HCC, and roughly 20% of HCCs have mutations in the β-catenin gene. More than 50% of HCC tumors display nuclear accumulation of β-catenin indicating that other factors may be involved such as aberrant methylation of the tumor suppressors APC and E-cadherin, inactivation of casein kinase and GSK-3β, or increased secretion of Wnt ligants [4]–[5].

There has been increasing interest in the role of liver cancer stem cells (LCSC) in tumorigenesis, tumor progression, invasion and metastases. The cancer stem cell theory suggests that a tumor is comprised of a heterogeneous population of cells that form a distinct cellular hierarchy. Recent studies have provided convincing evidence that these cells do exist in solid tumors of many types including, brain, breast, colorectal, liver, pancreas and prostate cancers. In 2006, two different groups isolated a CD133+ subpopulation from HCC cell lines and described higher proliferative and tumorigenic potential, consistent with stem cell properties. CD44 was also found as an important marker used in combination with other stem cell markers to better define the surface phenotype of LCSC. It has been demonstrated that CD133+ and CD90+ cells co-expressing CD44+ are more aggressive than those expressing CD133 or CD90 alone [13]–[14].

The chemical agents used to target Wnt-/β-catenin pathway are at the membrane, cytosol and transcription factor levels [5]. The small molecular agent FH535 is a dual inhibitor of peroxisome proliferator-activated receptor (PPAR) and β-catenin/TCF/LEF. FH535 has been shown to inhibit proliferation of HCC and hepatoblastoma cell lines and its specificity on inhibition of β-catenin/TCF/LEF activity was illustrated in hepatoblastoma cell line HepG2 [15].

The aim of this study was to determine if FH535 can inhibit the activation of β-catenin-regulated genes by endogenous and ectopically expressed β-catenin in the HCC cell lines Huh7, Hep3B and PLC and liver cancer stem cells (LCSC). The specificity of FH535 on inhibition of β-catenin via TCF/LEF activation was assayed in dual luciferase reporter transfected in LCSC and in HCC cells. Proliferation, cell cycle, and other targeted genes and proteins were assayed.

## Materials and Methods

### 2.1 Cell culture

The HCC cell line Huh7 [16] was a gift from Dr. Guangxiang Luo (University of Alabama – Birmingham). The HCC cell lines Hep3B and PLC were purchased from American Type Culture Collection (ATCC; Manassas, VA, USA). Hep3B and PLC both express HBV surface antigen, and Huh7 express hepatitis delta antigen. The Hep3B is p53 negative, the PLC has reduced p53 expression, and Huh7 has increased p53 protein level [17]–[18]. These cell lines were cultured in Dulbecco's Modified Eagles Medium (DMEM; Invitrogen, Carlsbad, CA, USA) supplemented with 10% fetal bovine serum (FBS), glutamine and penicillin/streptomycin. The liver cancer stem cells (LCSC; Catalog # 36116-43, CelProgen, San Pedro, CA, USA) were expanded up to and used within the first 4 passages in CelProgen complete growth media with 10% FBS in extra-cellular matrix (ECM) coated flasks (CelProgen). Flow cytometry profile and tumorigenicity of the LCSC are shown in Figures S1 and S2. Other cell lines were cultured in standard plastic wares without ECM coating. Cells were cultured in NuAire incubator (Plymouth, MI, USA) at 37°C with 5% CO_2_.

### 2.2 Chemicals

FH535, XAV939 and LiCl were purchased from Sigma-Aldrich (St. Louis, MO, USA). Methyl-^3^H-thymidine (2 Ci/mM) was from MP Biomedicals (Costa Mesa, CA, USA). The X-Treme Gene 9 and Turbofect transfection reagents were from Roche (Indianapolis, IN, USA) and Thermo Scientific (Waltham, MA, USA), respectively. The propidium iodide-based cell cycle analysis kit was from GenScript (Piscataway, NJ, USA). All the other chemicals were from Sigma-Aldrich (St. Louis, MO, USA).

### 2.3 Plasmids

The TOPFlash luciferase reporter gene, which contains 3 copies of a TCF/LEF binding site, and FOPFlash luciferase reporter, identical to TOPFlash except the TCF/LEF sites have been mutated, were purchased from Addgene (Cambridge, MA, USA) [19]. The pRL renilla luciferase vector was from Promega (Madison, WI, USA). Expression vectors for wild-type β-catenin and βCatS37A were provided by S. Byers [20] and E3-pGL3, containing mouse alpha-fetoprotein enhancer E3 fused to pGL3-promoter, was described previously [21].

### 2.4 ^3^H-thymidine incorporation assay

This assay was performed as described previously by our published method and by other researchers [22]–[24]. Briefly, cells were seeded in 96 well plates at 1,000–5000 cell/well in 0.2 ml DMEM +10%FBS and treated in duplicate with different concentrations of FH535 for 72 h. The cells were pulsed with ^3^H-thymidine at 1 µCi/well for 4 h. Alternatively, cells were cultured at 2500 cells/well in 0.1 ml of medium for 24 h, followed by the simultaneous addition of FH535 (0–15 µM) and ^3^H-thymidine (1 µCi/well) to a final volume of 0.2 ml/well., followed by incubation for an additional 18 hours. In both situations, at the end of incubation, cells were washed and fixed in 0.3 ml of 10% trichloroacetic acid (TCA) for 12 min. After aspiration of TCA, the cells were lysed in 0.2 M NaOH/0.2% SDS for 40 min. ^3^H-thymidine incorporation was measured by scintillation counting in a Packard Scintillation Analyzer (Covina, CA, USA). We did not perform MTT assay for cell proliferation to compare with ^3^H-thymidine incorporation because there is color reaction between FH535 and the MTT assay reagent (thiazolyl blue tetrazolium bromide). The concentration of FH535 to cause a 50% inhibition of cell grown (IC_50_) was determined by the following method. No-drug data was used as 100% at the Y-axis, and from this a 50% inhibition on the Y-axis was determined. From the 50% value point, we draw a parallel line to the X-axis (the drug concentration axis) to identify the cross point on the concentration curve. From the cross point we draw a parallel line to the Y-axis, and find the cross point on the X-axis. This cross point on the X-axis is the IC_50_ point. All experiments were performed at least twice.

### 2.5 Transient Transfections and Dual Luciferase Assays

For transfections, Huh7, PLC and LCSC were plated at 3×10^5^ cells/well in 1 ml culture medium in 12 well plates; after 24 h, cells were co-transfected with TOPFlash/pRL vectors (DNA ratio 10∶1) or FOPFlash/pRL vectors (DNA ratio 10∶1) using X-treme Gene 9 (Roche, Indianapolis, IN, USA) transfection reagent following manufacturer's instruction. After 6 h, the medium was aspirated and cells were added with 1 ml of culture media containing different concentrations of FH535, DMSO alone (vehicle), and/or 10 mM LiCl. In all cases, the final concentration of DMSO was 0.08%. The amount of vehicle DMSO was held constant in drug treatments and the final concentration of DMSO in each treatment was the same and was designed to less than 0.1%. After 36 h, dual luciferase assays were done with the Promega Dual Luciferase Assay System (Madison, WI, USA) according to manufacturer's protocols. Luciferase activity was measured in the Lumat LB 9507 luminometer (Berthold Technologies, Oak Ridge, TN, USA). Briefly, the cells were washed once with sterile PBS, and lysed in 1× lysis buffer (Promega) (0.25 ml/well) for 15 min at room temperature. Ten µl of crude cell lysate (prepared according to Promega Protocol) was added to 50 µl of luciferase assay LARII substrate in a 12×75 mm polystyrene tube (Fisher Scientific, Pittsburgh, PA, USA) to measure the firefly luciferase activity, followed by addition of 50 µl of Stop and Glo substrate to measure the Renilla luciferase activity. The measured Renilla luciferase activity was used to normalize the measured firefly luciferase activity.

For co-transfections with β-catenin expression vectors, cells were plated at 1×10^5^ cells/well in 24-well plates. Cells were transfected with 340 ng Luciferase reporter plasmid, 170 ng β-catenin expression plasmid, and 10 ng pRL/well using the Turbofect transfection reagent (Pittsburg, PA, USA). After 5-6 h, FH535 (or DMSO vehicle control) was added. After an additional 36 h, cell extracts (prepared according to Promega protocol) were prepared and used immediately or stored at −80°C. All transfections were performed in duplicate and repeated at least two times. Luciferase assays were performed with cell extracts using the Dual Luciferase Assay System (Promega, Madison, WI, USA) according to manufacturer's protocols. Luciferase activity was measured in duplicate.

### 2.6 Cell cycle analysis

Huh7 cells were cultured in DMEM +10% FBS for 24 h. The cells were washed with serum free DMEM 3 times, then cultured in DMEM +0.1% FBS for 24 h for synchronization of the cells. The cells were then cultured in DMEM+10% FBS with different concentrations of FH535 for 24 h. The cells were harvested and stained with propidium iodide (PI) and analyzed by flow cytometry according to the GenScript protocol. LCSC were cultured in CelProgen complete growth medium and treated the same way as indicated above.

### 2.7 RT-PCR and Western analysis

#### RT-PCR

Cells were cultured in DMEM+10% FBS in 100 mm tissue culture dishes until ∼70% confluence and then treated with DMSO alone or increasing concentrations of FH535 in DMSO for 38 h. For RNA analysis, cells were harvested and cDNA was prepared as described [21]. Quantitative PCR was carried out with SYBR green using the Bio-Rad MyiQ thermal cycler with the following primers: Survivin (BIRC5, CAAGGAGCTGGAAGGCTGG and GTTCTTGGCTCTTTCTCTGTCC), Cyclin D1 (CCND1: GGATGCTGGAGGTCTGCGA and TAGAGGCCACGAACATGCAAGT), and β2-microglobulin (B2M: GACTTTGTCACAGCCCAAGATAG and TCCAATCCAAATGCGGCATCTTC).

#### Western Blot

Huh7 cells (5×10^5^) were cultured in DMEM+10%FBS in 100 mm tissue culture dishes for 72 h. After change with fresh medium, the cells were treated with 0, 2.5, 5, and 10 µM of FH535 for 38 h. DMSO (<0.1%) were used as vehicle control. Protein concentrations in the cell extracts were assayed with micro BCA protein assay kit according to provider's protocol (Thermo Scientific Pierce, Rockford, IL, USA). Specific antibodies were obtained from Cell Signaling Technology (Danvers, MA, USA). Western Blot band density was assayed with NIH ImageJ software (Bethesda, Maryland, USA), and normalized by β-actin. All the other procedures were performed as previously described [25].

### 2.8 Statistical analysis

All analyses were performed using the software SPSS version 20 (International Business Machines Corporation, Endicott, NY, USA). Data are presented as mean ± SE. One-way ANOVA followed by Tukey correction was used to compare means. The level of statistical significance was set at *p*<0.05.

## Results

### 3.1 FH535 inhibits transcriptional activation mediated by wild-type and constitutively active β-catenin

**Figure 1:**
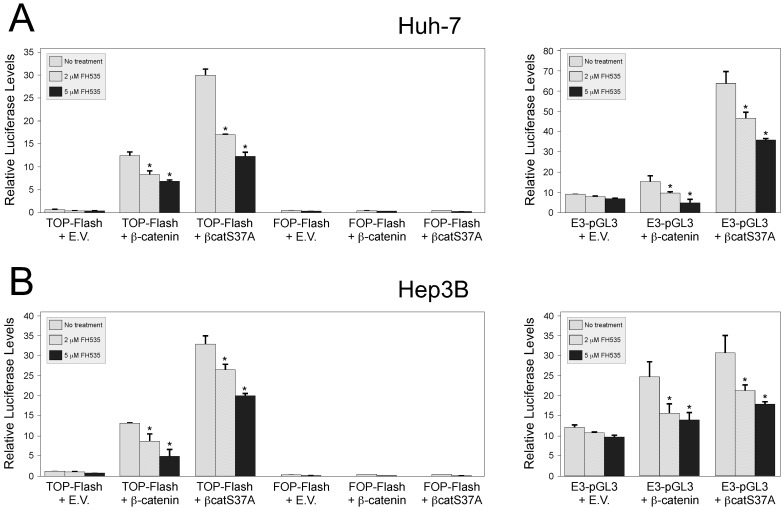
FH535 inhibits β-catenin dependent transcriptional activation in HCC cell lines. Huh7 (Panel A) and Hep3B (Panel B) HCC cells were transfected with the luciferase reporter genes TOPFlash (left panels), which contains three TCF binding sites, or E3-pGL3 (right panels), which contains the AFP enhancer element E3 that has a highly conserved TCF site. Cells were additionally co-transfected with an expression vector that contained no insert (empty vector control, E.V.), wild-type β-catenin (β-catenin), or a constitutively active form of β-catenin (βcatS37A). Renilla luciferase was used to control for variations in transfection efficiency. Six hours after the addition of DNA, cells were treated with DMSO alone (no treatment) or increasing amounts of FH535. After 48 hours, luciferase levels were determined; firefly luciferase was normalized to renilla. In both cell lines, FH535 inhibited β-catenin-dependent activation of target genes. * *P*<0.05. The experiment was done twice with similar results.

FH535 has been shown to block signaling through endogenous β-catenin in several cell lines, including the hepatoblastoma cell line HepG2 [15]. To further explore this regulation and to test whether FH535 could block ectopic β-catenin, co-transfections with β-catenin expression vectors and the TCF4-dependent luciferase reporter vector TOPFlash were performed in the human HCC cell lines Huh7 and Hep3B (Fig. 1). In both cell lines, co-transfected wild-type β-catenin expression vector increased luciferase activity from TOPFlash nearly 15-fold compared to cells co-transfected with the empty vector (E.V.) control. This β-catenin-dependent increase was inhibited by FH535 in a dose-dependent manner. β-catenin is often mutated in various cancers, including HCC. One natural mutation changes the serine at position 37; this altered form of β-catenin is resistant to degradation by the APC complex and thus has higher stability. To test whether this form of activated form of β-catenin could also be blocked by FH535, an expression vector for βCatS37A, in which the serine at position 37 has been changed to an alanine, was co-transfected with TOPFlash. As expected, βCatS37A-mediated transactivation of TOPFlash was significantly higher than transactivation by wild-type β-catenin. However, in both cell lines, βCatS37A-mediated transactivation was significantly inhibited by FH535. As controls, cells were also co-transfected with FOPFlash, which is identical to TOPFlash except that the TCF4 sites have been mutated and therefore no longer responsive to β-catenin; FOPFlash was not activated by wild-type β-catenin or βCatS37A as shown in Figure 1.

TOPFlash contains three consensus TCF4 binding motifs that confer responsiveness to β-catenin. To test whether FH535 could also block β-catenin-mediated transactivation of a TCF4 motif in the context of a natural regulatory region, co-transfections were performed with E3-pGL3. E3 is a ∼340 bp fragment that contains alpha-fetoprotein (AFP) enhancer element E3, one of three enhancers that control hepatic expression of the mouse AFP gene. E3 contains binding sites for multiple factors, including Foxa/HNF6, C/EBP, orphan nuclear receptors, and TCF4 [26]–[27]. We recently showed that this enhancer is regulated by β-catenin in cells and transgenic mice [21]. E3-pGL3 was transactivated by β-catenin and to a greater extent by βCatS37A (Fig. 1). However, this transactivation by both wild-type and S37A forms of β-catenin was blocked by FH535 in a dose-dependent manner.

### 3.2 FH535 inhibits β-catenin-mediated transcriptional activation in LCSC

**Figure 2:**
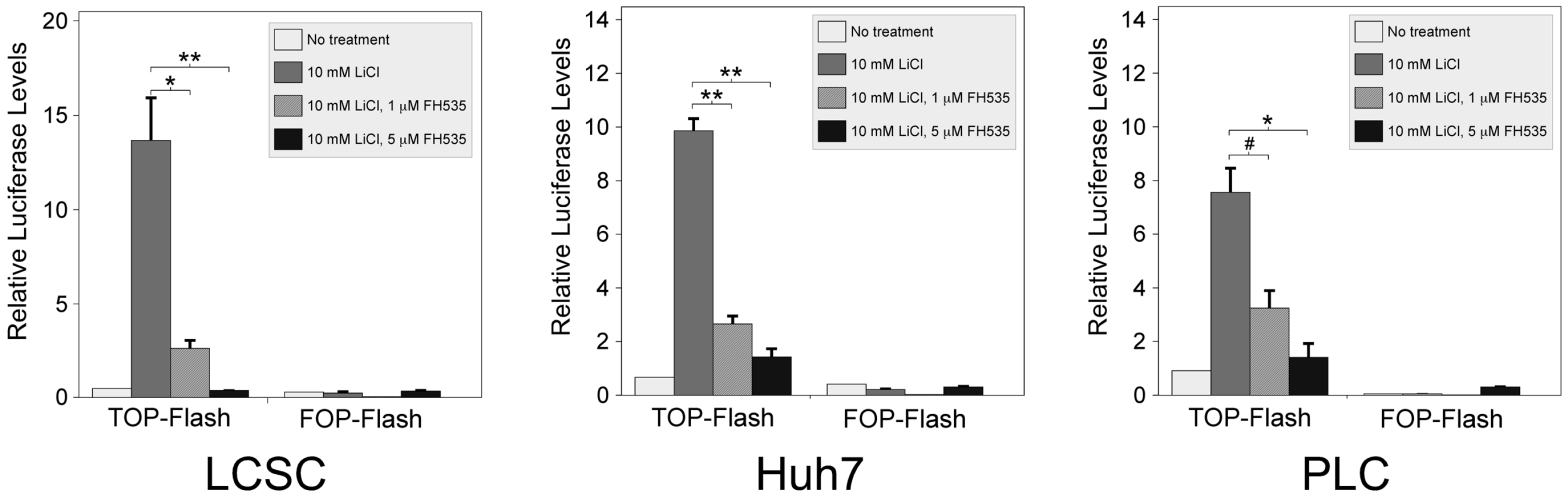
FH535 inhibits TOPFlash activation in LCSC and HCC cell lines. LCSC (left panel), Huh7 (middle panel) and HPLC (right panel) cells were co-transfected with TOPFlash or FOPFlash luciferase reporter genes along with renilla luciferase. After 6 hours, cells were left untreated (no treatment) or treated with LiCl alone or LiCl with increasing amounts of FH535. LiCl is a known activator of β-catenin. After an additional 36 hours, cells were harvested and luciferase levels were determined; firefly luciferase was normalized to renilla. TOPFlash activity was highly induced in all three cell populations; this activation was inhibited by FH535. The negative control FOPFlash showed minimal response to LiCl or FH535. TOPFlash inhibition by FH535 was more robust in LCSC than in either HCC cell line. * *P*<0.003, # P<0.001. The experiment was done twice with similar results.

Previous studies have shown that β-catenin signaling is elevated in EpCAM positive cells with LCSC properties [28]. We previously described that CD133+, CD44+, CD24+ LCSC aggressively form tumors when small numbers of these cells are injected into nude mice [29]. To test the ability of FH535 to inhibit β-catenin in these LCSCs, transient transfections were performed with TOPFlash. As controls, TOPFlash was also transfected into the HCC cell lines Huh7 and PLC (Fig. 2). In all three populations, untreated cells exhibited low luciferase levels. When treated with the GSK-3β inhibitor LiCl, which leads to endogenous β-catenin activation [30], TOPFlash activity increased dramatically. FH535 effectively blocked LiCl-mediated activation of TOPFlash in a dose-dependent manner. Interestingly, this inhibition was more robust in LCSC than in either HCC cell line. As a control, transfections were also performed with FOPFlash, which is no longer responsive to β-catenin. As expected, luciferase activity in FOPFlash-transfected cells was neither increased by LiCl nor inhibited by FH535.

### 3.3 FH535 inhibits proliferation of LCSC and HCC cell lines

**Figure 3:**
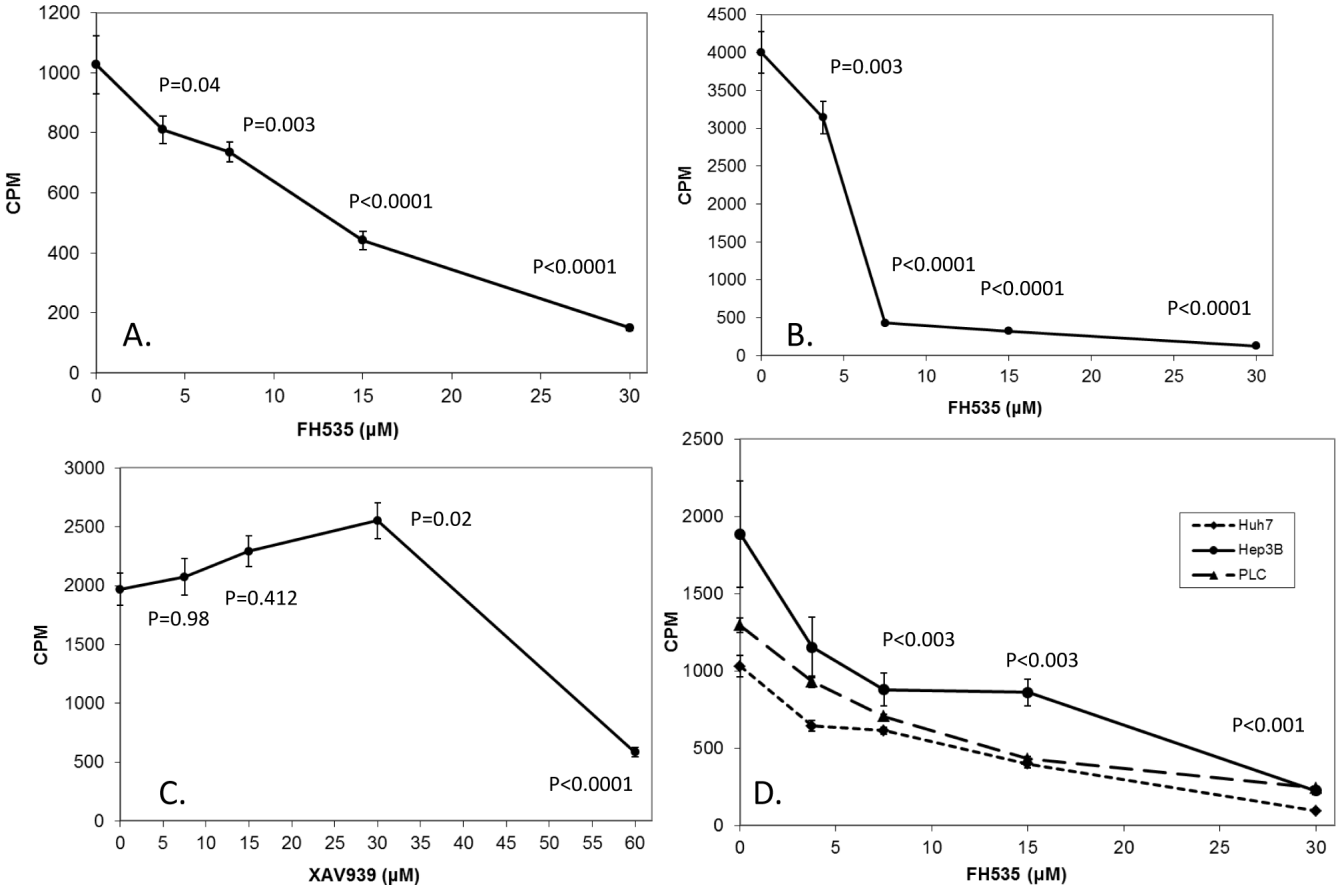
FH535 inhibits proliferation of LCSC and HCC cell lines. Cells were seeded in 96-well plates in 0.2 ml of media as described below for 72 hours, followed by the addition of ^3^H-thymidine at 1 µCi/well for 4 hours. Incorporation of ^3^H-thymidine was determined by scintillation counting. In panels A, B and D, the final concentration of DMSO in each well was 0.05%; in panel C, the final DMSO concentration in each well was 0.1%. (***A***) LCSCs were plated at 1000 cells/well in DMEM with 10% FBS along with DMSO alone or with increasing amounts of FH535. (***B***). LCSCs were plated at 5000 cells/well in DMEM with 1% FBS with DMSO alone or with increasing concentrations of FH535. (***C***). LCSCs were plated in DMEM with 10% FBS at 1000 cells/well with DMSO alone or increasing concentrations of XAV939. (***D***). Huh7, Hep3B and PLC cells were plated in DMEM with 10% FBS at 1000, 2500, and 5000 cells/well, respectively, with DMSO alone or increasing concentrations of FH535. *P* values are for all the three cell lines treated with FH535 are compared to controls. The experiment was done twice with similar results.

Numerous studies have demonstrated that β-catenin plays an important role in proliferation during normal development and in cellular transformation in many tissues, including the liver. Liver development is impaired in the absence of β-catenin, and mutations that activate the β-catenin pathway are found in about 1/3 of HCC [4]-[5]. Furthermore, the growth of adult liver progenitor stem cells (oval cells) can be inhibited by blocking the β-catenin pathway. Since our data indicated that FH535 can block β-catenin-mediated transcriptional activation, we also tested whether proliferation of LCSC and HCC cell lines was affected by this compound. LCSC were cultured in the presence of 10% or 1% serum and with between 5 µM and 30 µM FH535 for 72 hours, and cell proliferation was monitored by ^3^H-thymidine incorporation (Figs. 3A and 3B, respectively). Proliferation decreased with increasing amounts of FH535, with a more dramatic reduction observed in cells grown in the presence of lower serum; the concentration of FH535 to cause a 50% inhibition of cell grown (IC_50_) was 13.8 µM for cells grown in 10% serum and 5.1 µM for cells grown in 1% serum. This inhibition was more potent than that seen with XAV939 (IC_50_ = 55 µM), which inhibits tankyrase, thus stabilizing axin and promoting β-catenin degradation (Fig. 3C) [31]. FH535 also blocked proliferation of HCC cells at concentrations that were similar to that seen with LCSC (IC_50_ of 10.9 µM, 9.25 µM and 6.6 µM for Huh7, PLC and Hep3B, respectively; Fig.3D). To confirm that FH535 indeed inhibited cell proliferation and did not lead to increased cell death, FH535 and ^3^H-thymidine were added simultaneously to Huh7 cells, which were then cultured for 18 h. In this scenario, we observed a significant inhibition of proliferation at 2.5, 5, 10 and 15 µM of FH535 treatment as compared to control (p<0.05, n = 6), with FH535 at 15 µM causing a 41% inhibition (Figure S3). This data indicates that FH535 is inhibiting cell proliferation rather than increasing cell death.

### 3.4 FH535 induces cell cycle arrest in the HCC cell line Huh7 and in LCSC

**Figure 4:**
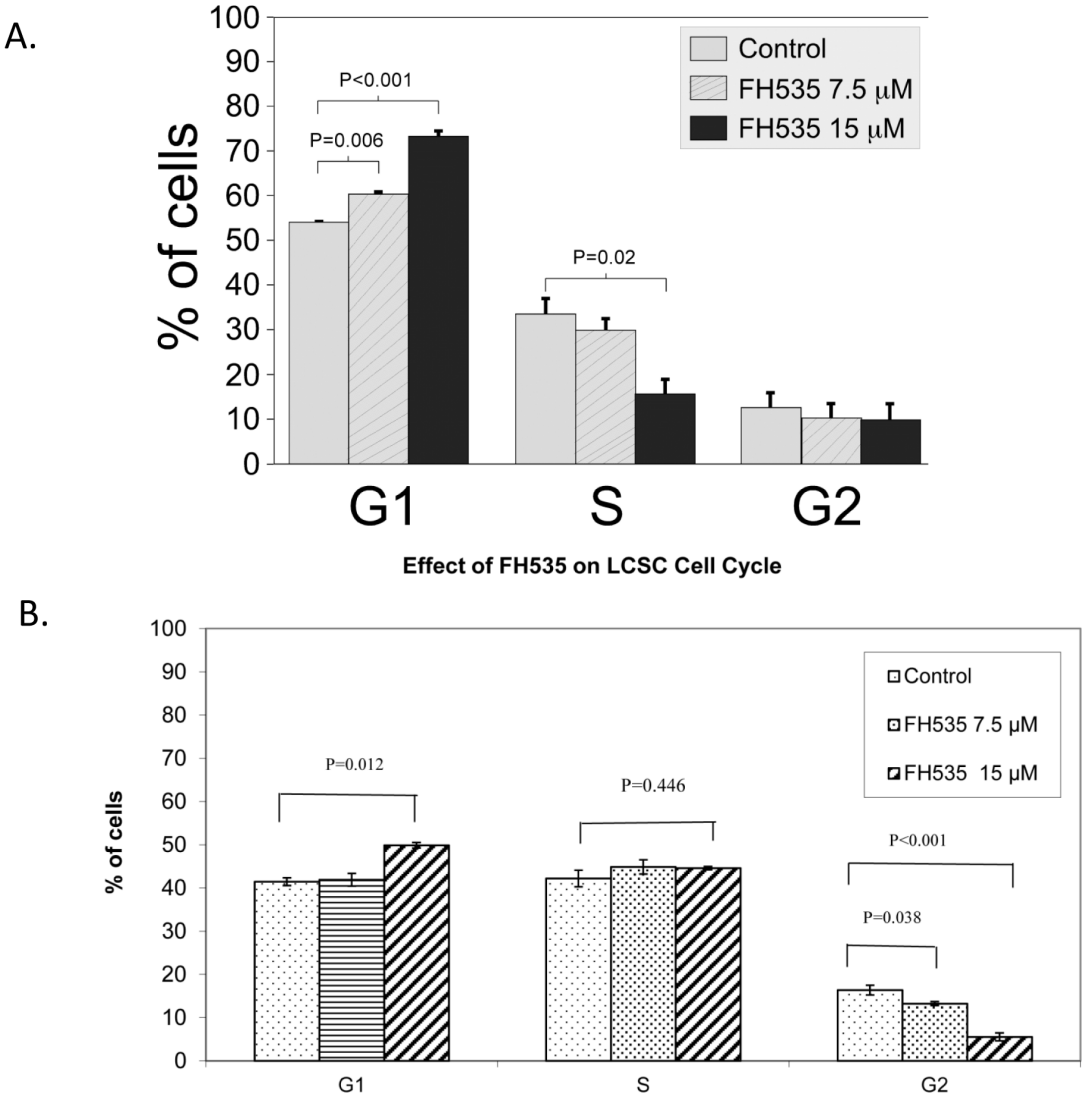
FH535 alters cell cycle progression in Huh7 and LCSC cells. **A**. Huh7 cells were cultured in DMEM +10%FBS for 24 h. The cells were washed with serum free DMEM 3 times, then cultured in DMEM +0.1% FBS for 24 h for cell synchronization. Cells were then cultured in DMEM+10% FBS along with different concentrations of FH535 for 24 h. The cells were harvested and stained with propidium iodide (PI) and analyzed by flow cytometry according to the GenScript protocol (Piscataway, NJ, USA). Treatment with FH535 increased the percentage of cells in G1 and decreased the percentage of cells in S phase. The experiment was done twice with similar results. **B**. LCSC cells were cultured in CelProgen complete LCSC culture medium for 24 h. Cells were then washed with serum free CelProgen medium 3 times and cultured in CelProgen Medium +0.1% FBS for 24 h for synchronization of the cells. The cells were then returned to CelProgen Complete Medium +10% FBS with different concentrations of FH535 for 24 h. Cell cycle was assayed as per Huh7 described above.

The ability of FH535 to inhibit cell proliferation prompted us to investigate the cell cycle distribution following treatment. Huh7 cells were synchronized by growth in 0.1% FBS for 24 hours and then cultured in the presence of 10% FBS and with no FH535 or FH535 at 7.5 µM and 15 µM. After 24 hours, cells were harvested and DNA content was analyzed by propidium iodide staining. In the presence of FH535, there was a statistically significant increase in the number of cells in G0/G1 and a corresponding decreased in the percentage of cells in S phase compared to cells grown in the absence of FH535 (Fig. 4A). The number of cells in G2 was not significantly altered by FH535. In addition, there was no sub-G1 peak detected by flow cytometry, indicating that FH535 was not promoting apoptosis at the concentrations being use (see Figure S4). We also did cell cycle analysis in LCSC after FH535 treatment and found FH535 at 15 µM significantly caused G1 phase arrest in LCSC (P = 0.012). FH535 also significantly reduced G2/M phase in the LCSC after 24 h of 7.5 µM and 15 µM FH535 treatment (P = 0.038 and P<0.001 respectively), no significant S phase inhibition was observed in LCSC (p = 0.446) (Fig. 4B.). Our data are similar to previously published results and reflects β-catenin regulation of cell cycle is different in different cell types [32]–[33]. Cell cycle regulators (cyclins, CDKs and regulators) can vary in different cell types, which could lead to different responses after FH535 treatment. This may worth exploring in our future study.

### 3.5 Expression of β-catenin target genes cyclin D1 and Survivin is inhibited by FH535

**Figure 5:**
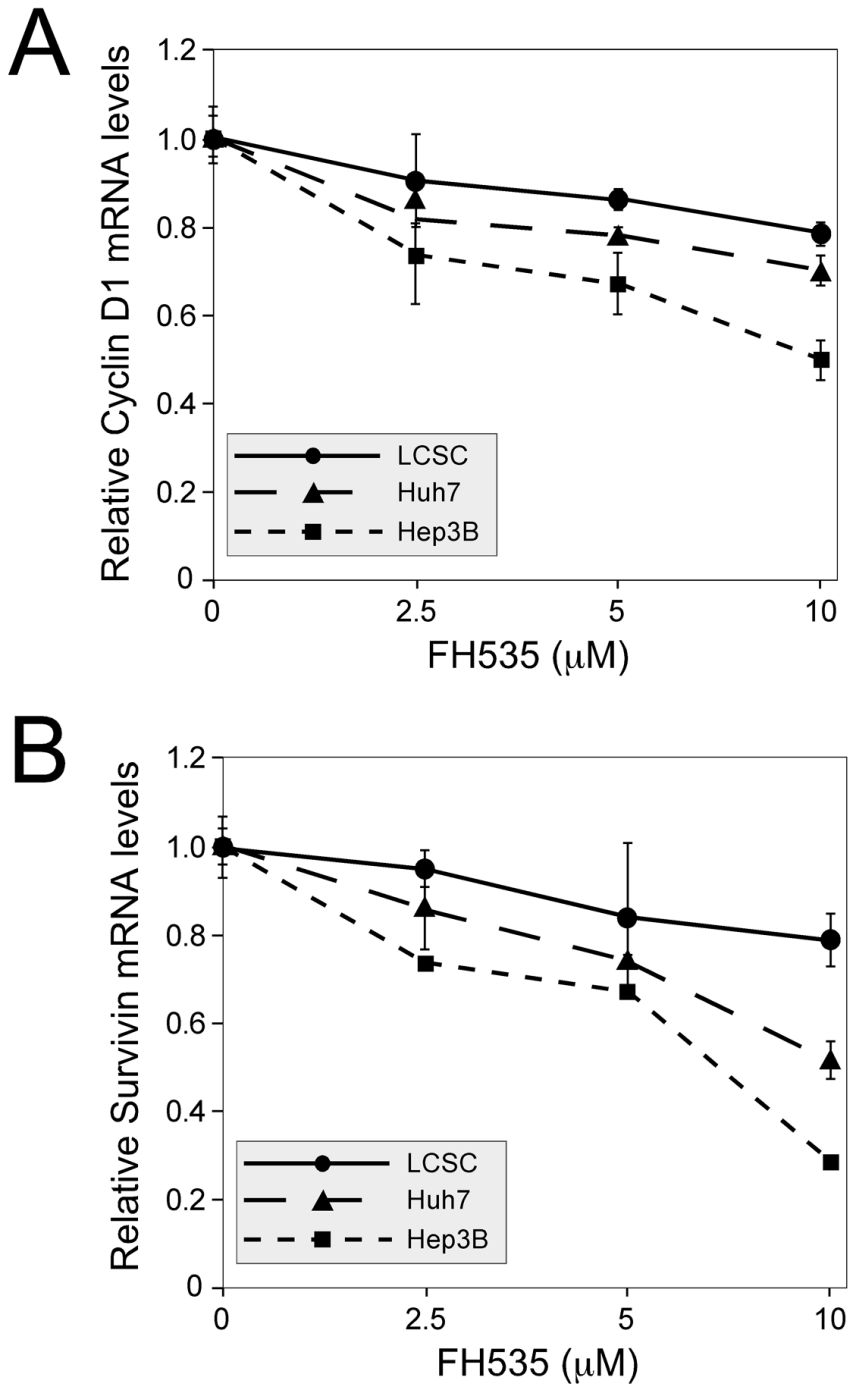
FH535 reduces cyclin D1 and survivin mRNA levels in LCSC and in HCC cell lines. LCSCs, Huh7 and Hep3B cells were treated with DMSO alone or increasing concentrations of FH535 for 38-time PCR for expression of Cyclin D1 (Panel A) or Survivin (Panel B). In both cases, mRNA levels were plotted relative to β_2_-microglobulin. The experiment was done twice with similar results.

**Figure 6:**
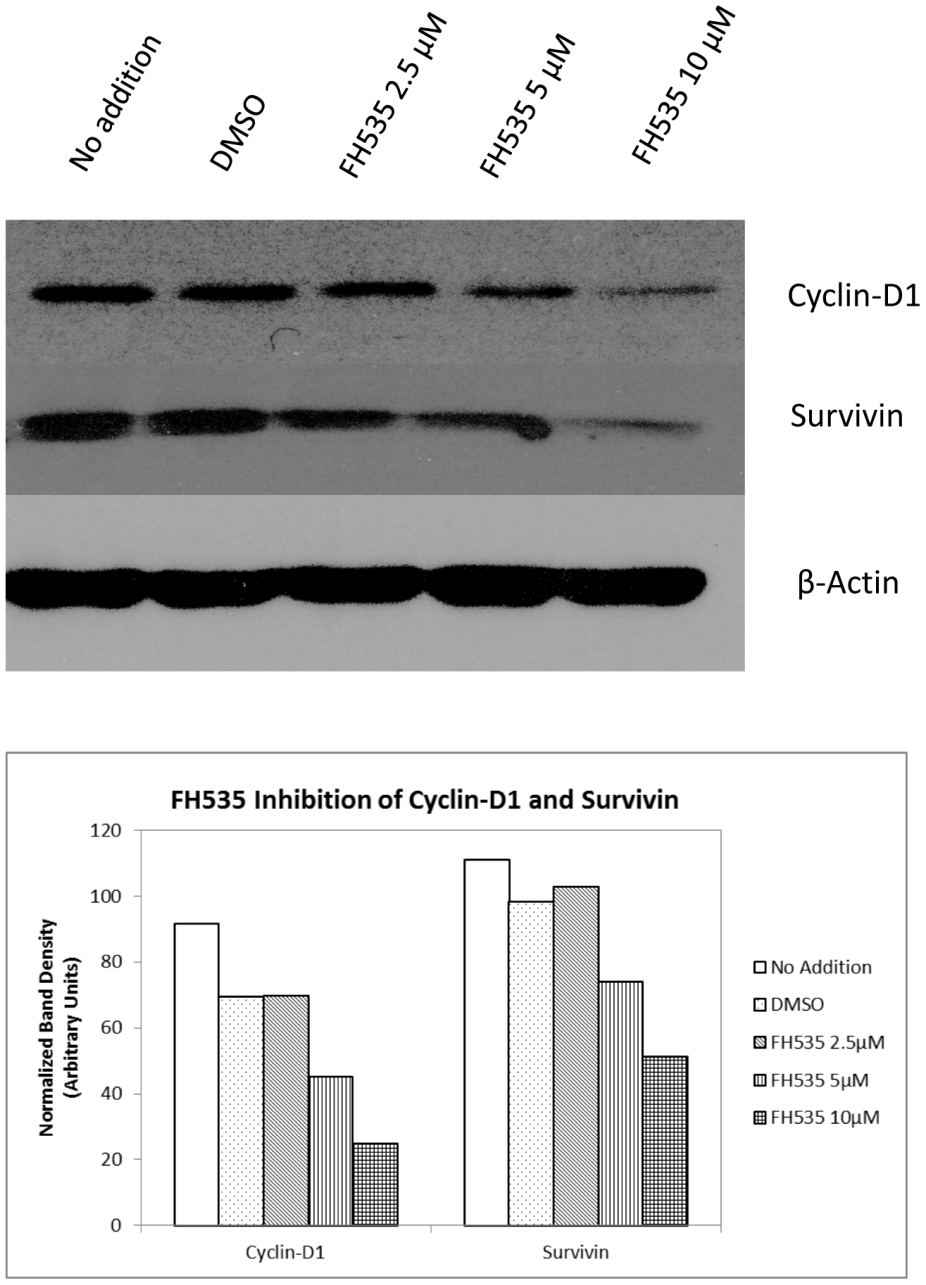
FH535 reduces cyclin D1 and Survivin protein levels in Huh7 cells. Huh7 cells were treated with DMSO alone or increasing amounts of FH535 for 38-PAGE, and transferred for Western analysis with antibodies against Cyclin D1, Survivin, and β-actin. The top of shows the western blot image; the bottom graph shows densitometric analysis of the western data. This densitometric analysis indicated that FH535 at 5 and 10 µM inhibited Cyclin D1 protein levels 28% and 64% respectively; FH535 at 5 and 10 µM inhibited Survivin protein levels 24% and 48% respectively. The experiment was done twice with similar results.

β-catenin controls cell proliferation and survival by regulating the expression of numerous targets genes. Two well-established targets are the genes encoding Survivin (Birc5) and Cyclin D1 (CcnD1). Survivin is an anti-apoptotic protein that also regulates progression through mitosis [34]; Cyclin D1 controls proliferation by activating the G1 kinases cdk4 and cdk6 [35]. Survivin and Cyclin D1 transcription are regulated through TCF elements in their promoter regions [36]. To test whether FH535 inhibits expression of these two β-catenin target genes, real-time RT-PCR was performed with LCSC and HCC cells that were treated with increasing amounts of FH535. Cyclin D1 and Survivin mRNA levels were reduced by FH535 in all three cell populations in a dose-dependent manner (Fig. 5). To confirm that this reduction in mRNA levels also led to lower protein levels, western analysis was performed using whole cell extracts from Huh7 cells. Both Cyclin D1 and Survivin protein levels were reduced in a dose-dependent manner, with the greatest reduction seen in the presence of 10 µM FH535 (Fig. 6.). Densitometric analysis indicated that FH535 at 5 and 10 µM inhibited Cyclin D1 28% and 64% respectively; FH535 at 5 and 10 µM inhibited surviving 24% and 48% respectively (Fig. 6).

## Discussion

In recent years, numerous signaling pathways have been implicated in hepatic carcinogenesis. The β-catenin pathway is essential in stem cells for self-renewal and maintenance of stem cell properties. Disruption of this balance results in both genetic and epigenetic changes, found in many cancers, including colon cancer and HCC [4]. In this study, we used FH535 as an inhibitor of the β-catenin pathway. This compound has been used previously to inhibit β-catenin expression in cells from colon and lung as well as in cells from hepatoblastoma and HCC [15]. In this report, the authors concluded that FH535 was toxic to a number of cell lines, including Huh7. However, their assays could not distinguish between toxicity and reduced cell proliferation. Our data indicates that FH535 does indeed inhibit cell proliferation; we did not directly measure toxicity.

FH535 inhibition of LCSC proliferation is of interest due to its potential therapeutic effect in chemo-resistant HCC. Our group and others have focused on strategies to inhibit the proliferation of LCSC and differences in resistance patterns with non-liver cancer stem cell lines *in vitro* and *in vivo*.

Despite numerous efforts, the etiology of HCC tumorigenesis, whether transformed cells originate from mature hepatocytes or stem/progenitor cells remains unclear. Stem cells are defined by their potential for self-renewal and by their ability to proliferate and differentiate into diverse cell types [37]. In recent years, studies have provided convincing evidence that these cells do exist in solid tumors of many types including, brain, breast, colorectal, liver, pancreas and prostate cancers [27]. In this study we have used LCSC that are 64.4%, 83.2%, 96.4% and 96.9% positive, respectively, for CD133, CD44, CD24 and Aldehyde A1 as determined by flow cytometry. These cells have been previously profiled not only by checking the LCSC markers but also by evaluating their tumorigenic potential using low cell numbers (using 2000 LCSCs instead of 100,000 HCC cells to generate tumors) and studying resistance to several drugs. We previously found that these LCSC have intermediate to high resistance to drugs compare to non- liver cancer stem cell lines using different inhibitors.

In this study, we found that FH535, LCSC inhibition of proliferation was affected by FBS concentration in the culture medium, suggesting that the PPAR pathway may be involved in LCSC proliferation as found in the human cancer cell line HCT116 [15]. This could be explained by a variety of fatty acids and their derivatives present in the FBS that are natural agonists to PPAR. It is possible the PPAR agonists suppress the inhibitory effects of FH535 in cell culture. Indeed, in HCT116 cells, FH535 inhibition of β-catein/TCF-dependent luciferase reporter genes was five times stronger in serum-free medium than in media containing 10% FBS. The ability of FH535 to inhibit tumor growth was dramatically increased when 10% FBS was replaced with 10% BSA [15]. Lysophosphatidic acid was found to be an effective PPAR agonist that could reverse FH535 induced inhibition of HCT116 growth [15]. However, the potential function of PPAR in LCSC is beyond the scope of this study and needs further investigation. Recently, FH535 was found to be the most potent drug among several other Wnt/β-catenin inhibitors on human biliary tract cancer cells cultured in serum-free medium [38]. Our study found that FH535 is much more potent than XAV939 in 10%FBS DMEM. This may be related to the PPAR inhibition potential of FH535. Our study found that FH535 inhibited HCC cell lines Huh7, Hep3B and PLC proliferation, indicating that Wnt/β-catenin signaling plays an important role not only in LCSC but also in HCC.

FH535 inhibition of LCSC and HCC proliferation was illustrated by its ability to inhibit β-catenin/TCF/LEF-dependent luciferase reporter activity. To our knowledge, this is the first report on the ability of FH535 to inhibit β-catenin/TCF/LEF activity in LCSC and in HCC cell lines. Previously, Handeli and Simon reported that FH535 inhibits β-catenin/TCF/LEF activity in the HepG2 cell line, which was mistakenly labeled as HCC by these authors [15]. For over thirty years this cell line was considered HCC by numerous investigators. Lopez et al., who initially isolated these cells, recently concluded that HepG2 cells should in fact be considered a hepatoblastoma cell line [39]. Further studies will be needed to investigate how FH535 inhibition of β-catenin influences LCSCs and HCCs. As shown here, cyclin D1 and Survivin expression are inhibited by FH535. Survivin is an anti-apoptotic protein that also regulates progression through mitosis [26], whereas Cyclin D1 controls proliferation by activating the G1 kinases [35]. Real-time RT-PCR and Western analysis confirmed that the expression of these target genes was evident at the mRNA and protein level. Our preliminary data indicate that FH535 treatment does not alter CD133, CD13 and EPCAM expression in LCSC and HCC cell lines (data not shown). Further analysis of these and other stem cell markers are warranted.

In conclusion, our data show that FH535 is a potent inhibitor of the Wnt/β-catenin pathway in LCSCs and HCC cell lines. Whether its ability to inhibit PPAR also affects the growth of LCSCs and HCC cells will require further investigation. Further studies will also be needed to investigate the *in vivo* efficacy and toxicity of FH535 on HCC xenografts in an animal model. The role of combination therapy using FH535 with other anti-HCC drugs and the possibility of finding cross-talk of Wnt/β-catenin pathway with other signaling pathways should be investigated.

## Supporting Information

Figure S1 LCSC at passage 2 were used for flow cytometry assays. Cells were washed with 1× PBS and then treated with 2 ml 1× PBS + 0.12% EDTA in T25 flask and incubate at 37°C for 3 min. The cells were scraped to suspend with cell scraper. Trypsin was not used to prevent degradation of CD133, CD44, CD24 and ALDH markers. After centrifugation, the cells were dissolved in 200ul of PBS +0.12% EDTA and FITC-conjugated antibodies, (or negative control antibodies) were added at 1∶500 dilution, followed by incubation at 30 min on ice. The stained and unstained cells were then analyzed for CD133, CD44, CD24 and ALDH using flow cytometry. FITC-CD133 antibody was from Cel-Progen, FITC-CD44, FITC-CD24 and FITC-ALDH1 was ordered from eBioscience (San Diego, CA, USA). In these LCSCs, the CD133^+^ populations was 64.4% (A), the CD44^+^ population was 83.2%, the CD24^+^ population was 96.4% and the ALDHA1^+^ population was 96.9% (D).
(TIF)

Figure S2 Female NOD/SCID mice (NOD.CB17-prkdc∧SCID/NCrSD, 4–5 week old) were purchased from Harlan Animal Research Laboratory (Indianapolis, IN, USA), housed and maintained in our Division of Laboratory Animal Resources animal facility. Mice received filtered air, sterile water and irradiated food *ad libitum*. Tumors were generated by harvesting first passage of LCSC cells (CelProgen Catalog number 36116-43, San Pedro, CA) that were cultured in CelProgen Liver Cancer Stem Cell Growth Media with Serum, from mid-log growth phase and trypsinized with 0.05% Trypsin/EDTA (Invitrogen). Cells were then washed and resuspended in a 50% mixture of Matrigel (BD Biosceince, San Diego, CA, USA) in CelProgen Liver Cancer Stem Cell Growth Media (serum free) to final cell number of 20,000 cells/ml. A volume of 0.1 ml of the cell suspension (2000 cells) was injected subcutaneously at the right flank of each mouse. The mice were checked for tumor growth every other week and mouse weight was measured. Tumors were found 28 days after inoculation in all the 3 tested mice. When the tumor sizes reached 940–1020 mm^3^, the mice were euthanized by CO_2_. The tumors were isolated and fixed in 10% Formalin for 48 h and subsequently changed to 70% ethanol. The tumors were paraffin embedded, cut to 5 µm sections and hematoxylin and eosin stained for histological analysis. A: H&E 200× This photomicrograph depicts a tumor growing in sheets of disorganized, haphazardly-oriented and pleomorphic cells that attest to its poor differentiation. Brisk mitotic activity is present and areas of necrosis are seen, implying rapid growth. B: H&E 400× This tumor is composed of cells with pleomorphic, vesicular nuclei that have prominent nucleoli with occasional macronucleoli. There are large numbers of mitoses, including abnormal forms with quadripolar spindles, reflecting very high proliferative activity.
(TIF)

Figure S3 Huh7 cells were plated to 96 well plates at 2500 cells/well in 0.1 ml culture medium and cultured for 24 h. The following day, ^3^H-thymidine and varying concentrations of FH535 were added simultaneously to the designated wells and cultured for 18 h. ^3^H-thymidine incorporation was assayed as indicated in Materials and Methods. *: p = 0.007 as compared to control; **: p<0.001 as compared to control (n = 6).
(TIF)

Figure S4 Flow cytometry indicates that a sub-G1 peak is not observed in Huh7 cells treated with FH535, indicating that FH535 does not increased apoptosis as judged by DNA fragmentation. A. Control (DMSO alone). B. FH535 at 7.5 µM. C. FH535 at 15 µM.
(TIF)
