# Supplementary figures and images for: Targeting the Wnt/β-Catenin Signaling Pathway in Liver Cancer Stem Cells and Hepatocellular Carcinoma Cell Lines with FH535
Source: PLoS One. 2014 Jun 18;9(6):e99272. doi: 10.1371/journal.pone.0099272 (PMC4062395; doi:10.1371/journal.pone.0099272)

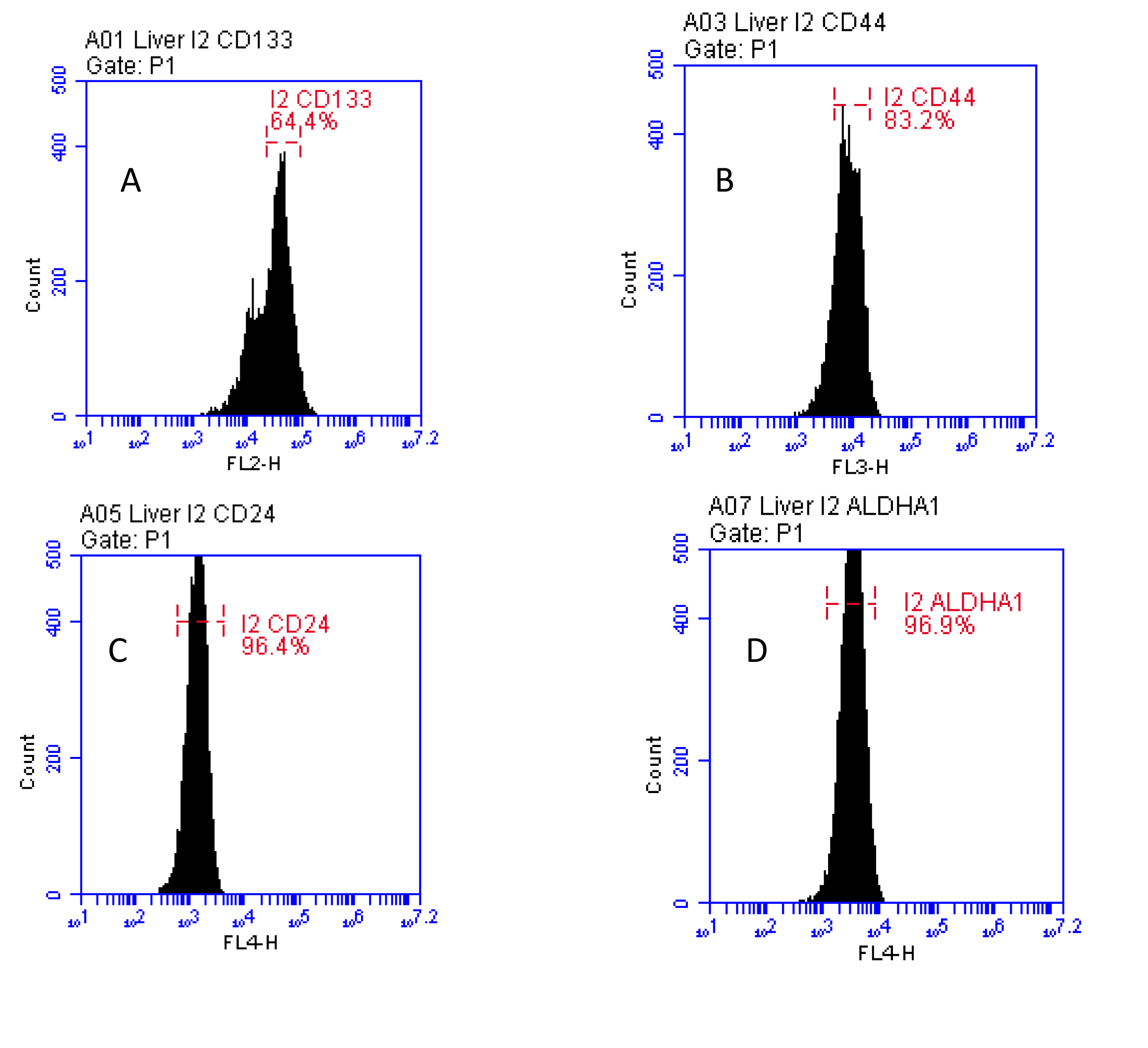

Supplement: Figure S1 — LCSC at passage 2 were used for flow cytometry assays. Cells were washed with 1× PBS and then treated with 2 ml 1× PBS + 0.12% EDTA in T25 flask and incubate at 37°C for 3 min. The cells were scraped to suspend with cell scraper. Trypsin was not used to prevent degradation of CD133, CD44, CD24 and ALDH markers. After centrifugation, the cells were dissolved in 200ul of PBS +0.12% EDTA and FITC-conjugated antibodies, (or negative control antibodies) were added at 1∶500 dilution, followed by incubation at 30 min on ice. The stained and unstained cells were then analyzed for CD133, CD44, CD24 and ALDH using flow cytometry. FITC-CD133 antibody was from Cel-Progen, FITC-CD44, FITC-CD24 and FITC-ALDH1 was ordered from eBioscience (San Diego, CA, USA). In these LCSCs, the CD133+ populations was 64.4% (A), the CD44+ population was 83.2%, the CD24+ population was 96.4% and the ALDHA1+ population was 96.9% (D). (TIF) [file pone.0099272.s001.tif]

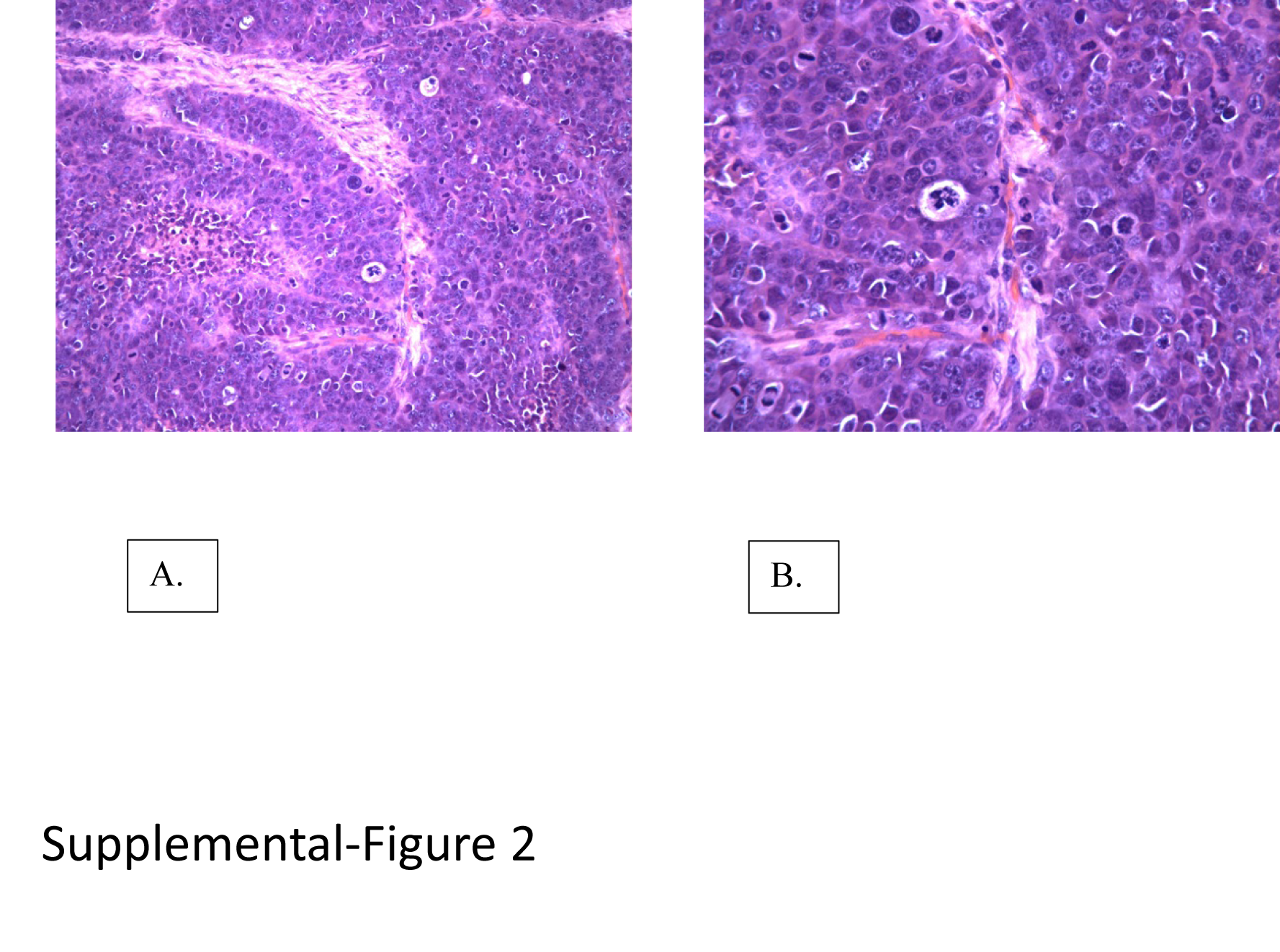

Supplement: Figure S2 — Female NOD/SCID mice (NOD.CB17-prkdc∧SCID/NCrSD, 4–5 week old) were purchased from Harlan Animal Research Laboratory (Indianapolis, IN, USA), housed and maintained in our Division of Laboratory Animal Resources animal facility. Mice received filtered air, sterile water and irradiated food ad libitum. Tumors were generated by harvesting first passage of LCSC cells (CelProgen Catalog number 36116-43, San Pedro, CA) that were cultured in CelProgen Liver Cancer Stem Cell Growth Media with Serum, from mid-log growth phase and trypsinized with 0.05% Trypsin/EDTA (Invitrogen). Cells were then washed and resuspended in a 50% mixture of Matrigel (BD Biosceince, San Diego, CA, USA) in CelProgen Liver Cancer Stem Cell Growth Media (serum free) to final cell number of 20,000 cells/ml. A volume of 0.1 ml of the cell suspension (2000 cells) was injected subcutaneously at the right flank of each mouse. The mice were checked for tumor growth every other week and mouse weight was measured. Tumors were found 28 days after inoculation in all the 3 tested mice. When the tumor sizes reached 940–1020 mm3, the mice were euthanized by CO2. The tumors were isolated and fixed in 10% Formalin for 48 h and subsequently changed to 70% ethanol. The tumors were paraffin embedded, cut to 5 µm sections and hematoxylin and eosin stained for histological analysis. A: H&E 200× This photomicrograph depicts a tumor growing in sheets of disorganized, haphazardly-oriented and pleomorphic cells that attest to its poor differentiation. Brisk mitotic activity is present and areas of necrosis are seen, implying rapid growth. B: H&E 400× This tumor is composed of cells with pleomorphic, vesicular nuclei that have prominent nucleoli with occasional macronucleoli. There are large numbers of mitoses, including abnormal forms with quadripolar spindles, reflecting very high proliferative activity. (TIF) [file pone.0099272.s002.tif]

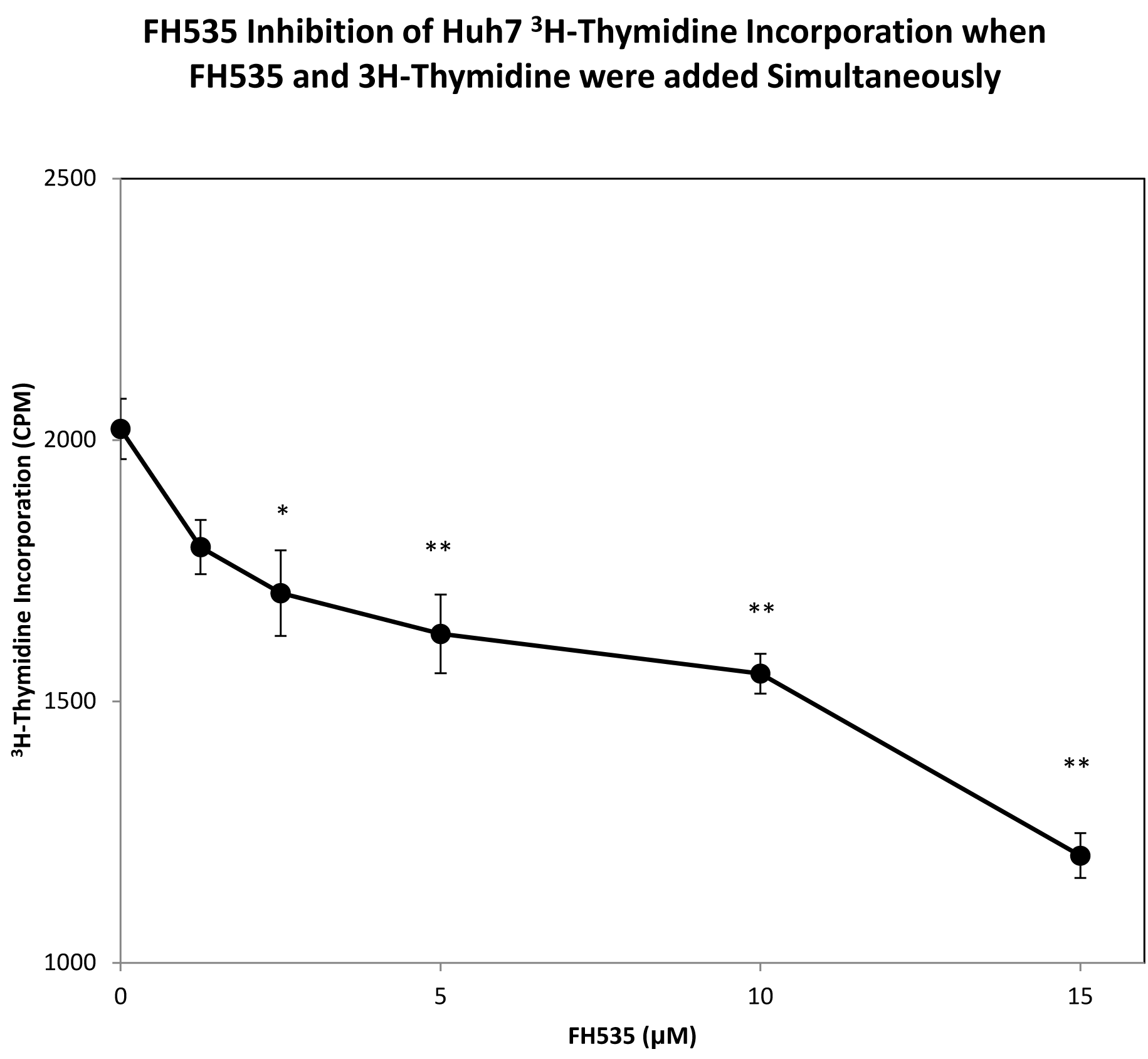

Supplement: Figure S3 — Huh7 cells were plated to 96 well plates at 2500 cells/well in 0.1 ml culture medium and cultured for 24 h. The following day, 3H-thymidine and varying concentrations of FH535 were added simultaneously to the designated wells and cultured for 18 h. 3H-thymidine incorporation was assayed as indicated in Materials and Methods. *: p = 0.007 as compared to control; **: p<0.001 as compared to control (n = 6). (TIF) [file pone.0099272.s003.tif]

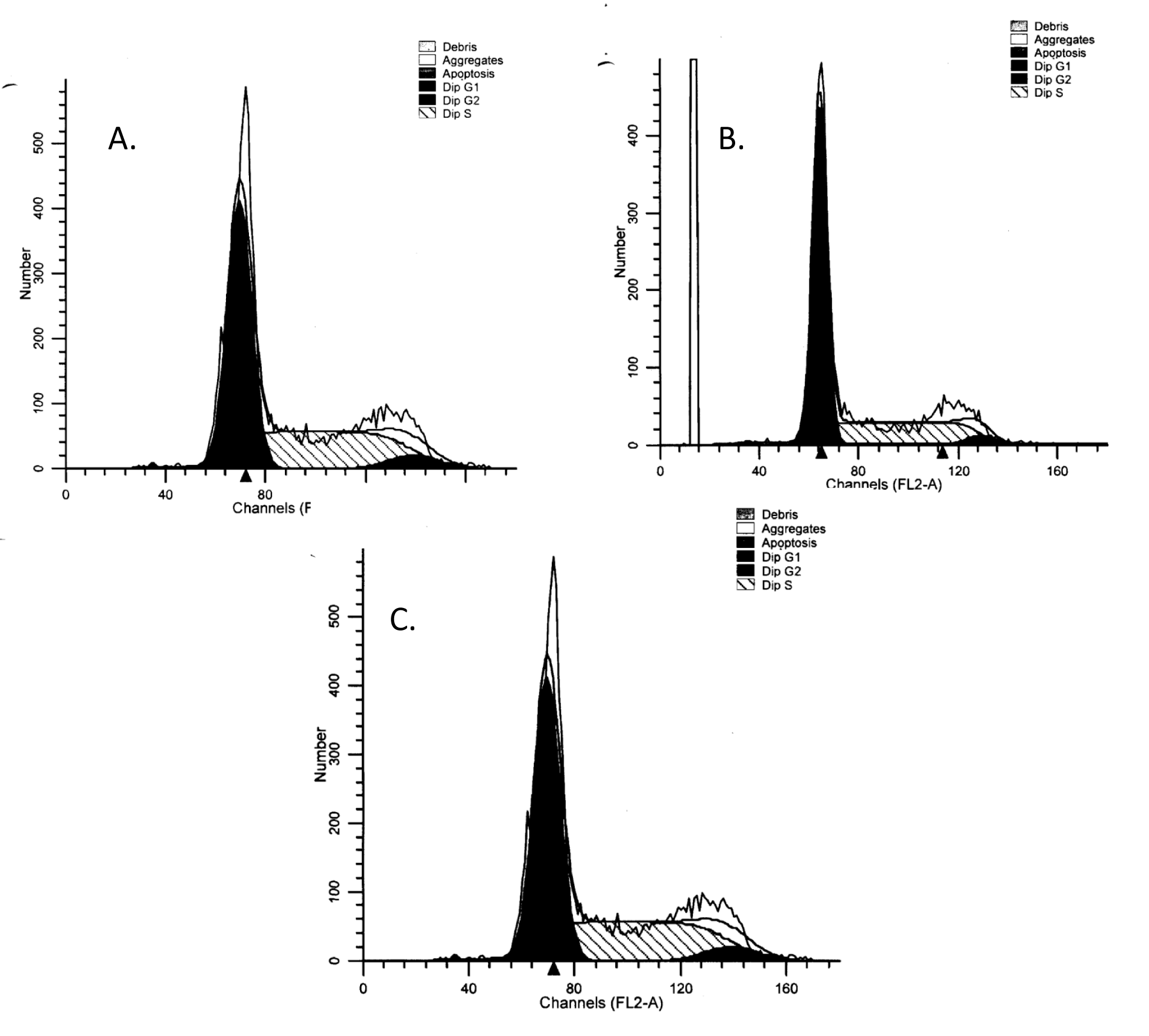

Supplement: Figure S4 — Flow cytometry indicates that a sub-G1 peak is not observed in Huh7 cells treated with FH535, indicating that FH535 does not increased apoptosis as judged by DNA fragmentation. A. Control (DMSO alone). B. FH535 at 7.5 µM. C. FH535 at 15 µM. (TIF) [file pone.0099272.s004.tif]
